# Depth Correction of TOF-SIMS Depth Profiling Images Using the Total Ion Count Images

**DOI:** 10.3390/biom15091237

**Published:** 2025-08-27

**Authors:** Melanie A. Brunet, Brittney L. Gorman, Mary L. Kraft

**Affiliations:** 1 Department of Chemical and Biomolecular Engineering, University of Illinois Urbana-Champaign, Champaign, IL 61801, USA; melanie.brunettorres@nih.gov; 2 Center for Biophysics and Quantitative Biology, University of Illinois Urbana-Champaign, Champaign, IL 61801, USA; brittney.gorman@pnnl.gov

**Keywords:** TOF-SIMS depth profiling, depth correction, endoplasmic reticulum-plasma membrane junctions

## Abstract

Depth profiling time of flight secondary ion mass spectrometry (TOF-SIMS) enables imaging the distributions of unlabeled metabolites within cells. When depth profiling TOF-SIMS is performed on intact cells, the 3D renderings produced by stacking and rending the individual depth profiling images are distorted along the z-axis, which complicates image interpretation. Here we describe an approach for correcting the z-axis distortion in 3D TOF-SIMS depth profiling images of cells. This approach uses the total ion images collected during TOF-SIMS depth profiling to create a 3D morphology model of the cell’s surface at the time when each depth profiling image was acquired. These morphology models are used to correct the z-position and height of each voxel in the component-specific 3D TOF-SIMS images. We have applied this approach to 3D TOF-SIMS depth profiling images that show endoplasmic reticulum-plasma membrane (ER-PM) junctions in cells that are a simplified model of ER-PM junctions in neuronal cells. The depth corrected 3D image more accurately depicted the structure of the ER-PM junctions than the uncorrected image. Projection of the depth corrected 3D image on the model of the cell’s morphology facilitated visualization of the ER-PM junctions relative to the peaks, ridges and valleys on the surface of the cell. Thus, accurate component-specific 3D images may now be produced for depth profiling TOF-SIMS datasets. This approach may facilitate efforts to identify the lipids and other metabolites that reside in ER-PM junctions in neuronal cells and elucidate their roles in neuronal function.

## 1. Introduction

Various biomolecules are heterogeneously distributed between organelles within cells and changes in these intracellular distributions are correlated with disease. Consequently, much effort has been made to characterize the subcellular distributions of specific biomolecules in healthy and diseased cells. Many studies of biomolecule distribution have focused on specific proteins due to their important roles in cellular function and the availability of genetically encoded fluorescent protein reporters and fluorescent microscopy techniques that enable their visualization. Though harder to visualize, studies have revealed that cholesterol and specific types of lipids are unevenly distributed within cell membranes [1,2,3,4,5,6] and between the membranes of different organelles [7,8], and changes in these distributions are associated with disease [9,10,11,12,13,14,15,16,17].

Characterizing the subcellular distributions of cholesterol and specific lipid families is challenging because these metabolites cannot be expressed as fusions with genetically encoded reporters (i.e., fluorescent proteins). Fluorophore-labeled lipid analogs may be metabolically incorporated into cells, but the metabolism and intracellular distributions of these lipid analogs may differ from the native species. For this reason, time-of-flight secondary ion mass spectrometry (TOF-SIMS), an imaging mass spectrometry method that identifies unlabeled molecules according to their distinct masses, has been used to characterize the subcellular distributions of cholesterol and specific lipid species [17,18,19,20,21,22,23]. TOF-SIMS uses a focused beam of primary ions to interrogate the sample and may achieve <1 µm lateral resolution and <5 nm depth resolution. When the primary ion beam is scanned across the sample, it fragments the superficial molecules within its focal area. The charged molecular fragments, which are called secondary ions, are directed to a mass spectrometer that measures each secondary ion’s mass, which can be used to identify the molecule that produced it. The counts of each secondary ion species detected at each pixel are also recorded. This information is used to generate maps that show the distribution of each secondary ion species, and thus the molecule that produced it, on the sample.

Although TOF-SIMS only detects the molecules on the sample’s surface each time the primary ion beam is scanned across the sample, component distribution inside a sample may be investigated by performing TOF-SIMS in a depth profiling mode. In TOF-SIMS depth profiling, multiple cycles of image acquisition using the primary ion beam, followed by the removal of a layer of material from the sample using a sputtering ion beam, are performed at the same position on the sample. This produces a series of secondary ion images that each show the same sample location but progressively deeper within it. The depth profiling images for each secondary ion species may be stacked in the order that they were acquired and rendered to produce a three-dimensional (3D) image showing the distribution of the parent molecule within the sample [24,25,26,27,28,29,30,31,32,33,34]. Unfortunately, when SIMS depth profiling is performed on a contoured sample such as an intact cell, the 3D renderings are distorted along the z-axis [24,25,27,31,32,33,34]. This distortion occurs because each TOF-SIMS depth profiling image becomes a flat plane in the 3D image, and these flat planes do not match the contoured surface of the sample. Importantly, the z-axis distortion complicates interpreting the 3D renderings of intact cells.

We previously developed a depth correction strategy that corrects the z-axis distortion in 3D SIMS depth profiling images of cells without requiring measurements of cell topography or other correlated analyses performed on other instruments [25,34]. This depth correction approach uses the intensities of the secondary electrons collected at each pixel during SIMS depth profiling to create a 3D model of the sample’s surface morphology when each depth profiling image was acquired. Then these morphology models are used to shift the voxels in 3D SIMS depth profiling images to the correct height above the substrate (z-position) and adjust their dimensions in the z-direction. Unfortunately, secondary electron images are seldom acquired during TOF-SIMS depth profiling.

Here we report a depth correction strategy for TOF-SIMS depth profiling analyses of cells that lack secondary electron images. This approach uses the total ion count (TIC) images collected during TOF-SIMS depth profiling to obtain information about the sample’s height at each pixel. To develop this strategy, we used previously published TOF-SIMS depth profiling data showing a 70 μm × 70 μm × 40 nm region on a transfected human embryonic kidney (HEK) cell that expressed a voltage-activated potassium channel, Kv2.1, fused to GFP [26]. This recombinant GFP-Kv2.1 fusion protein induces the formation of endoplasmic reticulum-plasma membrane (ER-PM) junctions in HEK cells that resemble those found in cultured neuronal cells [35,36,37]. To permit detecting these ER-PM junctions, the GFP-Kv2.1-expressing HEK cell had been labeled with a commercial fluorescent stain of the endoplasmic reticulum that produces distinctive fluorine secondary ions during TOF-SIMS [26]. Using this dataset, we show that height information may be extracted from the TIC depth profiling images and used to produce depth corrected 3D images that more accurately depict the structure of the ER-PM junctions in the cell than the uncorrected 3D image. The topography information yielded by this depth correction approach allowed us to assess whether the endoplasmic reticulum-plasma membrane junctions were more abundant on the peaks or valleys on the surface of the cell. The ability to detect ER-PM junctions with TOF-SIMS and create depth corrected 3D renderings that more accurately show their structure could facilitate future efforts to identify the lipid species enriched at these sites and their roles in neuronal cell function.

## 2. Materials and Methods

### 2.1. TOF-SIMS Analysis

This work employed a previously published TOF-SIMS depth profiling dataset [26] acquired with a PHI nanoTOF II Parallel Imaging MS/MS instrument (Physical Electronics, Chanhassen, MN, USA) from a HEKcell (American Type Culture Collection, District of Columbia, USA) that expressed recombinant GFP-Kv2.1. This cell was attached to a silicon substrate and it had been labeled with ER-Tracker Blue White DPX^®^ (Thermo Fisher Scientific, Waltham, MA, USA). Multiple cycles of data acquisition followed removal of a layer of material from the sample were performed at the same location. Secondary ion images were acquired in unbunched mode with a 30 kV Bi_n_^q+^ liquid metal ion source operated with a current of 3 nA, a pulse width of 16 ns uncompressed and a repetition rate of 8300 Hz. 512 pixel × 512 pixel images of a 70 μm field of view were collected and tandem MS1 and MS2 data were acquired at each pixel. A 5 keV beam of Ar_2,5000_^+^ ions operated with 2.5 nA dc beam current was used to sputter material from an 800 × 800 µm region on the cell between the acquisition of depth profiling images. Depth profiling began at the cell surface and ended approximately 40 nm below it.

### 2.2. Ion Image Construction

Using PHI TOF-DR software (Version 2.0.0.25), depth profiling total ion count (TIC), m/z 19 count and m/z 77 count images were created using data collected during two raster cycles. The m/z 19 ions were identified as fluorine anions (F^−^) produced by the ER-Tracker stain based on their colocalization with less abundant, higher molecular weight fragment ions (m/z 167, pentafluorophenyl anion, C_6_F_5_^−^) that MS/MS analysis of a droplet of ER-Tracker Blue White DPX^®^ confirmed were characteristic of the ER-Tracker stain [26]. The m/z 77 ions were presumed to be hydrogen silyl anions (SiO_3_H^−^) because they were detected on the silicon substrate. Each depth profiling image, referred to as an image plane, was numbered in the order that it was acquired during the depth profile. 127 consecutive TIC images and F^−^ images were converted to grayscale and exported as bitmap files.

Subsequent image preprocessing was performed in MATLAB (RB2021b). Bitmap files for the TIC and F^−^ images were imported into MATLAB and a 3D matrix (512 pixel × 512 pixel × 127 image cycles) was compiled for each signal. The TIC images were aligned with MATLAB’s imregister function using the mean square error as the metric and fine-tuned with a custom algorithm that aligned every image plane to a user-selected feature. Lateral translations for the TIC image matrix were applied to the F^−^ and SiO_3_H^−^ count matrices. The F^−^ images were summed over a sliding window of 3 consecutive raster planes centered at the current plane. A 3 × 3 boxcar smoothing algorithm was applied to each image plane, and the matrix was normalized so that its maximum value equaled 1.

### 2.3. Masking

A threshold was applied to the matrix of substrate-specific m/z 77 ions presumed to be SiO_3_H^−^ to convert the elements with values below that threshold to zero. The nonzero elements remaining in the matrix were converted to one. Morphological operations were used to remove islands of ones or zeros surrounded by zeros or ones, respectively. Finally, the complement of this matrix was calculated, producing a binary mask matrix that contained ones at the pixels where cell was detected and zeros where the substrate was detected. If the resulting mask contained large regions where elements that corresponded to the substrate had values of 1, a manual masking procedure was used to convert those elements to zero. The resulting binary mask matrix was multiplied by the TIC matrix for the corresponding image plane so that the elements that represented pixels where the substrate was detected equaled zero.

### 2.4. Creation of 3D Models of Sample Morphology

**Figure 1:**
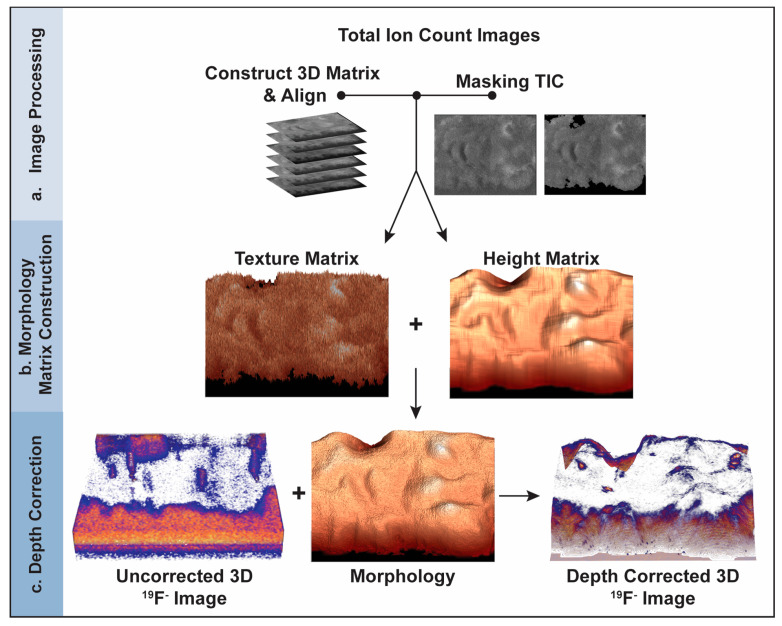
Schematic of the depth correction process using the intensities in the TIC images. (**a**) TIC image preprocessing. A 3D matrix that contains the pixel intensities for all the TIC images is constructed, each image plane is aligned, and the pixels corresponding to the substrate are masked, converting their intensities to zero. (**b**) 3D model construction for each image plane. A morphology matrix is created for each image plane by adding the texture matrix derived from the TIC for that image plane and the height matrix that accounts for the layers of material that were removed by each image-sputter cycle and the portion of the cell remaining after depth profiling. The morphology matrix is rendered to produce a 3D model of the sample when the corresponding image plane was acquired. (**c**) Depth correction of the 3D ^19^F^−^ image that locates the ER Tracker stain. The z-position of each voxel in the 3D component-specific image is shifted so it equals the height at the same x, y location in the morphology model for the corresponding image plane.

For each image plane in the depth profile, a morphology matrix was created and rendered to produce a 3D model of the cell’s morphology at the time that the corresponding image plane was acquired. Each morphology matrix was created by summing a height matrix that accounted for the height of the cell at each pixel in the image plane and a texture matrix that recreated the small projections and crevices on the surface of the cell (Figure 1).

The model of the cell’s morphology when the last image plane was acquired (image plane 127) was constructed first. The texture matrix for the last image plane was created by normalizing the masked TIC matrix for the last image plane to its highest value and multiplying the resulting matrix by the weighing constant for the last image plane (C_127_). The value of the weighting constant was empirically estimated by selecting a value, using it to create a morphology model, visually comparing the model to the cell morphology shown in the corresponding TIC image and adjusting the weighing constant’s value until good agreement was obtained. Based on this process, 0.02 was used for C_127_.

The height matrix for the last image plane was constructed as follows. First, a convolution kernel was used to average the masked TIC matrix for the last image plane in the depth profile. Next, the intensities of the pixels in the TIC image for the last plane that did not show the cell were zeroed, and all pixel intensities were normalized to the brightest pixel. Finally, the nonzero elements were scaled so that the highest value equaled the height of the tallest feature on the cellular material shown in the last image plane. The relative height of this tallest feature was estimated by ratioing the area occupied by cell material in the TIC image for the last image plane to that for the first image plane, and then multiplying this ratio by 127, the total image planes in the depth profile. The areas occupied by cell material for the first and last image planes were computed based on the number of pixels in the corresponding TIC image occupied by cell material, which was calculated by summing all elements in the binary mask matrix for that plane.

The model of the surface of the cell when the last image plane was acquired was completed by multiplying the height matrix for the last plane by 1−C_127_ and adding it to the texture matrix for the last image plane. MATLAB’s surf function was applied to the morphology matrix, producing a 3D surface representation of the cell’s morphology when that image plane was acquired.

The morphology models for the remaining image planes were constructed as described for the last image plane but with the following modifications. The height matrix for each remaining image plane was constructed by adding the height matrix from the last image plane to each layer of material that was sputtered from the cell during the subsequent image-sputter cycles. The layers of material removed from the sample by subsequent image-sputter cycles were created by assigning a relative height of one or zero to each pixel in the subsequent TIC images that showed the cell or substrate, respectively. The C_i_ value for each remaining image plane was estimated by scaling linearly from C_1_ to C_127_.

The morphology models for all image planes were adjusted so that the relative height of the cell at each pixel decreased each time an image plane was acquired. The difference between the relative height of each voxel in image plane 1 and the voxel at the same x, y location in image plane 2 were calculated. If this difference was negative, the height at that location in the morphology model for image plane 2 was reduced to the height at that location in image plane 1. Then the relative height of at each location in layer 2 was reduced by an amount proportional to the difference between the normalized total ion counts detected at the same location in both layers. This process was used to adjust the heights at every location in the morphology model for each succeeding image plane.

### 2.5. Depth Correction

The height at every position in each 3D morphology model was rounded to the nearest 1/5th of an image plane. The resulting height of each voxel was used to define the new z-position for the top of the corresponding voxel in a 3D secondary ion image. The height of the voxel in the secondary ion image was adjusted so that its bottom edge was adjacent to the top of the voxel in the subsequent image plane. The depth corrected 3D secondary ion matrices were visualized using the MATLAB function volview and 50% transparency.

## 3. Results

### 3.1. Morphology Model Construction

To correct the z-axis distortion in 3D TOF-SIMS depth profiling images of contoured samples, each voxel in the 3D image must be shifted to the z-position that corresponds to the height of the sample at that x, y location when the image plane it belongs to was acquired. For this reason, we sought to create a 3D model of the cell’s morphology for each image plane in the depth profile and to use the relative height at each x, y location on each model to adjust the z-position of the corresponding voxel in the 3D SIMS image. We hypothesized that, like the secondary electron images acquired during depth profiling with a NanoSIMS, the TIC images acquired during TOF-SIMS depth profiling contain information about the sample’s height that may be extracted for 3D model construction. Thus, our depth correction strategy for 3D TOF-SIMS depth profiling images is analogous to that we reported for 3D NanoSIMS images [25,34], but with modifications that allow the TIC images to be used in place of the secondary electron images.

Construction of the 3D models of the cell surface at the time each depth profiling image plane was acquired is described in detail in the Section 2. Briefly, the 3D model for each image plane is the rendering of the morphology matrix for that image plane. Each morphology matrix is the sum of a texture matrix that produces the cell’s textured appearance and a height matrix that accounts for most of the cell’s height (Figure 1). The texture matrix for each image plane was compiled by assigning a value of zero to the pixels in the TIC image for the corresponding image plane that did not show the cell, and then normalizing every non-zero pixel intensity by the highest intensity. The height matrix for each image plane was constructed by modeling the height of the cell material remaining on the substrate when depth profiling was complete and adding the layers of material that were sputtered from the cell during the subsequent image-sputter cycles. The height of the cell material that remained after depth profiling was estimated by zeroing the intensities of the pixels in the TIC image for the last plane that did not show the cell, normalizing all pixel intensities to the brightest pixel, and multiplying by a scaling constant. This scaling constant related the cell’s height after depth profiling to the height of the material removed by one image-sputter cycle. The layers of material removed from the sample by subsequent image-sputter cycles were created by assigning a relative height of one or zero to each pixel in the subsequent TIC images that showed the cell or substrate, respectively. Summation of the height of the cell material that remained after depth profiling and the layers removed by subsequent image-sputter cycles at each pixel produced the height matrix for the corresponding image plane.

Before the height and texture matrices for the same image plane could be summed to produce the morphology matrix for the corresponding plane, the proportion of the total cell height contributed by the height and texture matrices had to be determined for each image plane. For the first and last image plane, these proportions were empirically adjusted until the 3D model produced by applying MATLAB’s surf function to the morphology matrix matched the shape of the cell shown in the corresponding TIC image. A linear scale was used to set the proportions for the intermediate layers. Then for each image plane, the height and texture matrices were scaled by these proportions and summed, producing a preliminary morphology matrix for that plane.

Finally, the morphology models for every image plane were adjusted so that the relative height of the cell at each pixel decreased each time an image plane was acquired. First, the z-position of any voxel in image plane 2 that was greater (i.e., taller) than the corresponding voxel in image plane 1 was reduced to the height of the voxel in image plane 1. Then the overall shape of the cell material was preserved by reducing the z-position of each voxel location in image plane 2 by an amount proportional to the difference between the normalized total ion counts detected at the same location in both layers. This process was repeated for each succeeding image plane.

### 3.2. Evaluation of the Morphology Model

**Figure 2:**
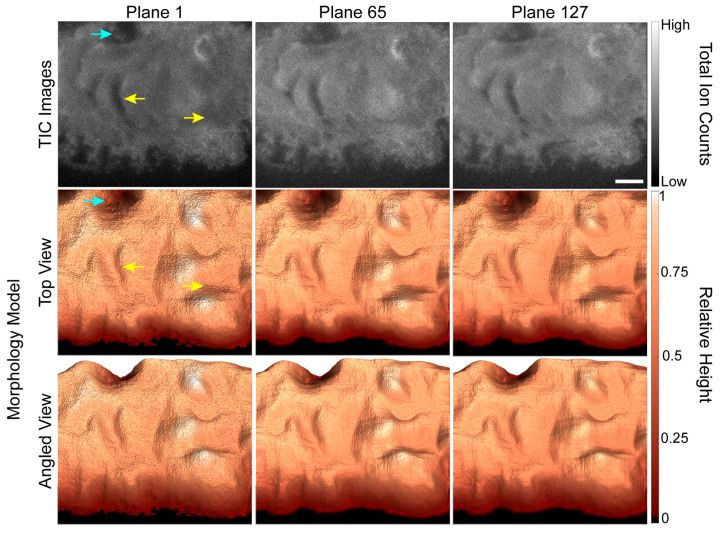
TIC TOF-SIMS depth profiling images of a GFP-Kv2.1-expressing HEK cell and the corresponding morphology models for the specified image planes. The morphology models replicated the cell’s general shape and most of its surface features, such as the hills and valleys indicated by yellow arrows. A few small discrepancies between the TIC image and surface model were identified, such as the valley that appears wider in the morphology model than the TIC image (blue arrow). Scale bar is 10 µm.

Because the accuracy of the depth corrected 3D SIMS images depends on the accuracy of the 3D morphology models, we evaluated how well each morphology model matched the shape of the cell shown in the TIC image for the corresponding plane. The first, 65th and 127th TIC images in the depth profile are shown in the first row of Figure 2, and two views of the corresponding morphology models are shown in the second and third rows. Each morphology model shown in Figure 2 has the same general shape and features as the cell shown in the corresponding TIC image. For example, the relatively deep valleys and adjacent hills (Figure 2, yellow arrows) on the cell that are shown in the TIC image for plane 1 are also present on the corresponding morphology model. Closer inspection of the images reveals two small discrepancies between the TIC images and morphology models. First, the width of the large depression near the upper left side of the cell (Figure 2, blue arrow) appears to be overestimated in the morphology models. Second, the extensions at the lower edge of the cell are present in the morphology model for the first image plane but not those for image planes 65 and 127. Both discrepancies were caused by the low contrast in the SiO_3_H^−^ secondary ion images that our automated masking procedure used to locate the pixels that did not show the cell. Nonetheless, the morphology models were largely consistent with features shown in the TIC images.

### 3.3. Secondary Ion Image Analysis

**Figure 3:**
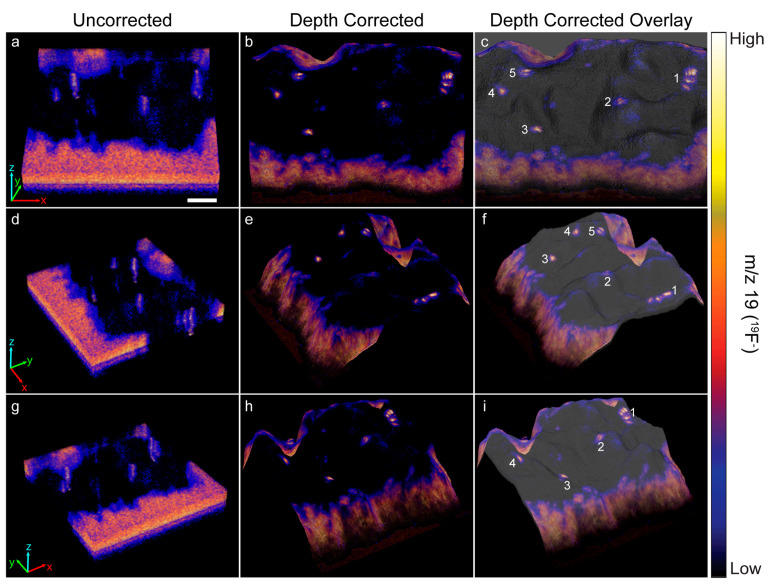
The uncorrected and depth corrected 3D ^19^F^−^ image that locate the ER-Tracker stain within the depth profiled HEK cell that expressed GFP-Kv2.1, and the overlay of the depth corrected ^19^F^−^ image on the morphology model are presented from three different perspectives. Local elevations in ^19^F^−^ within the cell denote ER-plasma membrane junctions. The five most prominant ^19^F^−^ hotspots are numbered to facilitate relocating them in each image. In the uncorrected ^19^F^−^ images (**a**,**d**,**g**) the ER-plasma membrane junctions were artifactually elongated because the image is distorted along the z-axis. Depth correction of the ^19^F^−^ images (**b**,**e**,**h**) placed each voxel at a z-position that more accurately represented its relative height within the intact cell. This repositioning eliminated the artifactual elongation of the ER-PM junctions, so they have a more compact structure in the depth corrected 3D ^19^F^−^ image. Overlay of the depth corrected 3D ^19^F^−^ image on the morphology model (**c**,**f**,**i**) enables visualizing the locations of the ER-plasma membrane junctions with respect to features on the surface of the cell. Scale bar is 10 µm.

**Figure 4:**
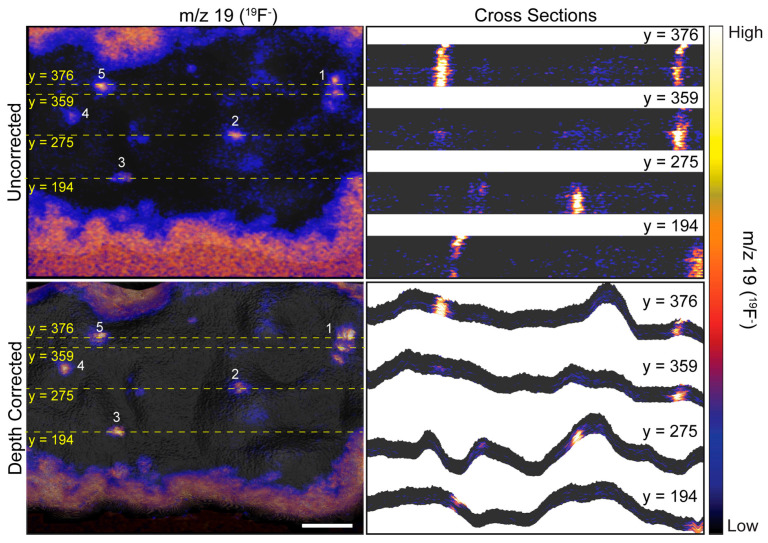
Cross sections taken at the indicated y-coordinate of uncorrected and depth corrected 3D ^19^F^−^ images. Dashed yellow lines show the position of each cross section on the image. Local elevations in ^19^F^−^ within the cell denote ER-PM junctions. Numbers 1–5 designate the same ^19^F^−^ hotspots that are labeled in Figure 3. The cross sections taken on the uncorrected 3D ^19^F^−^ image demonstrate the surface of the cell is incorrectly represented as planar, and the resulting distortion along the z-axis artifactually elongates the ER-PM junctions. The ER-plasma membrane junctions in the depth corrected ^19^F^−^ image have a more compact appearance because the cross sections follow the contours of the cell. Scale bar is 10 µm.

The heights encoded in each morphology model were used to depth correct the 3D ^19^F^−^ image that showed a ~70 μm × 70 μm × 40 nm region on top of a HEK cell that expressed recombinant Kv2.1 fused to GFP. Each voxel in the 3D image was shifted to the height at the same x, y location on the corresponding morphology model. Any empty space or overlap between voxels in adjacent image planes was removed by expanding or contracting the voxel in the z-direction, respectively. Figure 3 shows a top view and two side views of the uncorrected (Figure 3a,d,g) and depth corrected (Figure 3b,e,h) 3D ^19^F^−^ images and overlays of the depth corrected 3D ^19^F^−^ images on the morphology model for the cell (Figure 3c,f,i). Cross sections taken along the depth corrected 3D ^19^F^−^ image are presented in Figure 4.

The uncorrected and corrected 3D ^19^F^−^ images reveal local elevations in ^19^F^−^ within the cell. These ^19^F^−^ hotspots were produced by the ER-Tracker stain that labeled the endoplasmic reticulum. The close proximity of these ^19^F^−^ hotspots to the surface of the cell indicated they were ER-PM junctions. Note that the ^19^F^−^ hotspots appear to be elongated in the uncorrected 3D ^19^F^−^ images, whereas they have a more compact structure in the depth corrected images (Figure 3 and Figure 4). This elongation is an artifact of the z-axis distortion that occurs in the uncorrected 3D images, and depth correction rectified this distortion.

We examined the overlays of the ^19^F^−^ images on the morphology model (Figure 3) and their cross sections (Figure 4) to investigate whether the ^19^F^−^ hotspots that denoted ER-PM junctions were preferentially located on peaks or valleys on the cell surface. The prominent ^19^F^−^ hotspots are numbered in Figure 3 and Figure 4 to facilitate their identification. The overlay image (Figure 3) show that the cluster of three ^19^F^−^ hotspots (feature 1) was located on a ridge. The cross sections taken at y = 376 and y = 359 (Figure 4) confirmed that these three ER-PM junctions were positioned on a ridge, though that ridge was relatively small compared to other projections on the cell. The overlay images (Figure 3) and the cross section at y = 275 (Figure 4) indicate that the ER-PM junction denoted as ^19^F^−^ hotspot 2 was located slightly below the apex of a large hill. The ER-PM junction bisected by the y = 194 cross section (^19^F^−^ hotspot 3) was located near the top of a ridge that was adjacent to a much taller projection on the cell, and the ER-PM junctions denoted as ^19^F^−^ hotspots 4 and 5 also appear to be positioned slightly below a ridge. These observations suggest that the ER-plasma membrane junctions are likely to be positioned along the hills on the cell’s surface, and not at the apex of a hill or within a deep valley on the cell surface.

## 4. Discussion

Dysregulation of lipids and cholesterol is associated with many diseases, including those that afflict the brain [15,16,17]. This has motivated efforts to parse out the specialized functions of lipids and cholesterol in brain cells. Knowledge of the distributions of cholesterol and specific lipid species within neuronal cells and between distinct subcellular compartments, such as organelles, could yield new insights into the roles of cholesterol and lipids in brain function and disease. SIMS depth profiling is one of the few techniques that enable imaging the distributions of cholesterol and specific lipid species within a cell. However, the 3D images produced by stacking and rendering 3D SIMS depth profiling images of intact cells are distorted in the z-direction, which complicates image interpretation. In prior reports [25,34], we began to address this problem by using the secondary electron images acquired during SIMS depth profiling to create models of the cell’s morphology that are used to depth correct the 3D SIMS images. This approach could not be universally applied to TOF-SIMS depth profiling images of cells because secondary electron images often are not acquired during TOF-SIMS depth profiling. Here, we extended our strategy to 3D TOF-SIMS images of cells by using the total secondary ion counts in place of the secondary electron intensities to model the cell’s morphology. We demonstrated that the TIC images acquired during TOF-SIMS depth profiling may be used to create accurate models of the cell’s morphology at the time each depth profiling image plane was acquired, allowing each voxel in the 3D secondary ion image to be moved to the correct z-position. By applying this depth correction technique to 3D TOF-SIMS images of a GFP-Kv2.1-expressing HEK cell, we showed that the z-axis distortion that artifactually elongated the ER-PM junctions near the surface of the cell was eliminated. We also demonstrated that overlays of the depth corrected 3D images on the morphology models generated by our depth correction approach facilitated assessing the locations of the ER-PM junctions relative to features on the surface of the cell.

We aimed to create a depth correction strategy with greater applicability to SIMS depth profiling data acquired from cells than previously published depth correction strategies [24,25,27,31,33,34]. As mentioned above, cells are frequently depth profiled with TOF-SIMS and secondary electron images are not usually acquired, so we developed algorithms that use the TIC images to model the cell’s morphology. These morphology modeling algorithms do not use the detection of secondary ions from the substrate beneath the cell to define the z = 0 plane (e.g., the cell–substrate interface). Consequently, the entire cell does not need to be completely sputtered away during SIMS depth profiling to employ our depth correction strategy. This is advantageous when the components and structures of interest are not located near the bottom of the cell because sputtering away the entire cell is time consuming. Our morphology modeling algorithms also do not require the collection of topography measurements from the cell before or during SIMS analysis, which may be time consuming and challenging to acquire. Finally, because this depth correction strategy creates a separate model of the cell’s surface for every image plane acquired, it corrects any distortion in the z-direction produced by lateral variations in sputter rate without requiring knowledge of the sputter rate on that material. This is advantageous for studies of cells because the rate of sputtering on lipid-rich features such as membranes and lipid droplets may differ from that on protein and nucleic acid-rich structures such as ribosomes and the nucleus.

The research reported herein demonstrated the feasibility of using secondary ion images to depth correct 3D TOF-SIMS images. A comparison of our depth corrected images to the corresponding TIC images demonstrated that the depth corrected images reproduced the general shape of the cell’s surface. Thus, this depth correction technique produces 3D SIMS images that are more accurate than the uncorrected 3D images, which should improve image interpretation. However, the 3D SIMS images produced with this approach should not be used to measure height differences between features because our assessment of model accuracy was not quantitative. Future efforts should use correlated height measurements acquired with atomic force microscopy (AFM) to validate this approach. These validations should be performed using both negative and positive ion TOF-SIMS data acquired from a variety of different cell types and shapes. Whether the cell’s local composition, such as enrichment with lipids or proteins or the presence of different organelles, affects the accuracy of morphology model construction should also be investigated.

This depth correction strategy was developed for TOF-SIMS depth profiling images that lack correlated measurements of sample topography. Therefore, the sole metric for assessing the accuracy of the depth corrected 3D SIMS images is how well the features on the morphology models match those shown in the TIC images. This assessment allowed us to identify two discrepancies between our morphology models and the topology of the cell shown in the TIC images. Specifically, the large valley near the top left side of the cell was too large in the morphology model and the extensions that protrude from the lower edge of the cell were missing from the models for some image planes (Figure 2). However, because this accuracy assessment is subjective, the extent of these discrepancies and their effect on the accuracy of the depth corrected 3D TOF-SIMS images could not be estimated. Future research should focus on developing an assessment that quantifies how well each morphology model matches the sample topology shown at each pixel in the corresponding TIC image. The resulting numerical measures of model accuracy would allow the user to objectively select the values of the empirical variable that increase their models’ accuracies and could enable the development of algorithms that optimize these values. Future research should also aim to correlate the numerical measures of model accuracy with the relative height difference between the morphology model and the AFM measurements acquired to validate this depth correction strategy. Such correlations would enable estimation of the errors in the relative heights of various features in the models in the absence of AFM data.

The TOF-SIMS depth profiling dataset employed in this proof of concept study was acquired from a transfected human embryonic kidney (HEK) cell that expressed the Kv2.1 voltage-activated potassium channel fused to GFP [26]. In neuronal cells, the Kv2.1 channels involved in electrical signaling are dispersed within the plasma membrane, and the non-conducting Kv2.1 channels form clusters that establish stable junctions between the endoplasmic reticulum and plasma membrane [35,37,38,39]. These Kv2.1-stabilized endoplasmic reticulum-plasma membrane (ER-PM) junctions are trafficking hubs where membrane proteins are inserted into and removed from the plasma membrane [35,40,41]. Expression of recombinant GFP-Kv2.1 fusion protein in HEK cells induces the formation of ER-PM junctions that have biophysical properties and distributions like those in cultured neuronal cells [35,36,37]. Consequently, HEK cells expressing recombinant Kv2.1 are used as a simplified model to study the roles of ER-PM junctions in neuronal cell function. A combination of this transfected cell line and/or actual neuronal cells, ER-Tracker staining, TOF-SIMS depth profiling and the depth correction strategy reported herein might facilitate identification of the lipid composition at the ER-PM junctions. This approach might also be used to investigate the hypothesis that ER-PM junctions allow lipid exchange between the endoplasmic reticulum and plasma membrane in neuronal cells and identify any changes in the lipid composition of ER-PM junctions following ischemic events. Such studies could improve understanding of the Kv2.1-mediated ER-PM junction structure and its role in neuronal cell function and disease.

## 5. Conclusions

We have demonstrated that 3D TOF-SIMS depth profiling images acquired from contoured samples such as cells may be depth corrected in the absence of correlated height measurements. In this depth correction strategy, the TIC images collected during TOF-SIMS depth profiling are used to model the surface of the sample every time a depth profiling image is acquired. These morphology models are then used to adjust the z-positions and heights of the corresponding voxels in the 3D renderings of the depth profiling secondary ion images. In the absence of correlated height measurements, the accuracy of the morphology models had to be evaluated by comparison to the surface features shown in the TIC images. Such comparisons indicated this approach largely captured the morphology of the cell, though a few discrepancies could be identified. Nonetheless, the resulting depth corrected 3D TOF-SIMS images were more accurate than the uncorrected images, and they enabled assessment of the locations of the ER-PM junctions on the cell relative to the hills and valleys on the surface of the cell. Future methodology development should include validating this approach using correlated AFM measurements, a variety of different types of cells, and both negative and positive ion TOF-SIMS depth profiling datasets. The development of quantitative measures of morphology model accuracy should also be a priority.

The depth correction strategy reported herein is expected to facilitate the interpretation of TOF-SIMS depth profiling images acquired from intact cells. A combination of this depth correction strategy and ER-Tracker staining of neuronal cells could facilitate investigations of the lipid composition at ER-PM junctions and how this composition changes following ischemic events. The application of this depth correction approach to TOF-SIMS depth profiling data of neuronal cells could reveal the distributions of specific lipid species within healthy and diseased neuronal cells. Given the importance of lipid and cholesterol homeostasis in the brain, such research could help increase understanding of brain function.

## Data Availability

The TOF-SIMS depth profiling data that was used in this work is openly available at the Illinois Data Bank at https://doi.org/10.13012/B2IDB-5513643_V1.
